# Italian Nursing Research: A Bibliometric Analysis from 1980 to 2020

**DOI:** 10.3390/nursrep14040287

**Published:** 2024-12-09

**Authors:** Michela Luciani, Michela Barisone, Marco Bentivegna, Antonietta Fioremisto, Giulia Galeazzi, Marco Alfonso La Monica, Alessandra Musci, Davide Ausili, Alberto Dal Molin

**Affiliations:** 1 Department of Medicine and Surgery, Università degli Studi di Milano, 20126 Milano, Italy; michela.luciani@unimib.it (M.L.); antonietta.fioremisto@aorncaserta.it (A.F.); alessandra.musci@asst-brianza.it (A.M.); davide.ausili@unimib.it (D.A.); 2 Department of Translational Medicine, University of Piemonte Orientale, Via P. Solaroli 17, 28100 Novara, Italy; alberto.dalmolin@med.uniupo.it; 3 Dipartimento di Medicina Traslazionale, Università degli Studi del Piemonte Orientale “Amedeo Avogadro”, 13100 Vercelli, Italy; mbentivegna@aslto4.piemonte.it (M.B.); giuliagaleazziwork@gmail.com (G.G.); marcoalfonso.lamonica@asst-ovestmi.it (M.A.L.M.)

**Keywords:** bibliometric analysis, doctorate education, Italian nurses, nursing research, retrospective descriptive study

## Abstract

**Aim:** The aim of this study was to explore Italian nurses’ publications from 1980 to 2020. **Background/Objectives:** Several studies have been conducted internationally to assess nursing research output. In Italy, there are some older studies, but a comprehensive analysis of the Italian nursing scientific production after 2010 is needed. **Methods:** A bibliometric analysis was conducted through a retrospective descriptive study. All articles (n = 3423) published by Italian nurses (n = 2170) and indexed in Scopus were included, in accordance with the PRISMA guidelines. **Results**: Publication trends show a steady growth, with an increase in publications in journals with higher IFs. Most publications were focused on clinical research and used quantitative methods (n = 2473 articles (86.71%)). The most frequently conducted quantitative studies were observational studies (52.91%), followed by experimental studies (12.5%), instrumental studies (6.72%), and other methodologies (0.15%). Qualitative studies accounted for n = 318 articles (11.15%), and mixed-method studies accounted for n = 61 articles (2.14%). **Conclusions:** The overall improvement in Italian nursing research is due to the increase in the number of nurses with PhDs and academics in the country. More funding and nursing research positions are needed to further improve research.

## 1. Introduction

In nursing, research is fundamental for innovation in practice, and the quality of the first has direct implications for the latter [1]. By analyzing the research output of a discipline, it is possible to extrapolate its cultural and scientific evolution and its theoretical development [2,3]. Evaluating research outputs allows the scientific progress and cultural development of a discipline to be deduced [3,4]. Internationally, several studies were conducted to evaluate the global nursing research output, either with a general focus [5,6,7,8] or within a specific subspecialty, such as family nursing [9], oncology [10,11], or robotics [12]. A few bibliometric studies were also performed to evaluate the scientific production of single countries [13,14,15].

Italy has a strong public science base, even though the overall investment in research and innovation remains well below the European average [16]. In 2016, Italy produced almost 4% of the world’s 10% most-cited scientific publications, behind the United States, China, the United Kingdom, and Germany. As an example, looking at the specialized and topical area of focus artificial intelligence, Italy is the world’s fifth most-cited producer of scientific articles [17].

More specifically, Italy is one of the 20 most prolific countries for nursing research [7,8], and nine Italian nursing scientists were included in the 2020 ‘World’s Top 2% Scientists’ List’ [18]. This is most surprising considering that, at the time this study was designed, there were only 44 tenure-track nurse academics in Italy (assistant, associate, or full professors) [19]. Moreover, Italy is one of the high-income countries with the lowest gross domestic expenditure on Research and Development [20]. Finally, nursing only became a 3-year university bachelor’s degree in 2001, while Master of Science degrees in nursing were established in 2004 and doctoral programs in nursing were introduced in 2006 [21]. The first nursing professor was appointed in 2000 [22]. Therefore, in Italy, nursing research evolved along a different timeline compared, for example, to that of the United States, where in the 1950s there was already funding for nursing doctorate education and research had already been published by nurses [23].

A few studies investigated the scientific production of Italian nurse researchers during specific periods up until the 2000s: 1978–1997 [24], 1998–2001 [25], 1998–2003 [26], and 2003–2009 [27]. Later, more focused bibliometric analyses were conducted. Some focused on academia and investigated the impact of PhDs in nursing on scientific production from 2006 to 2015 [28] and the scientific activity of full-time nursing academics [22]. Some focused on evaluating research in nursing practice [29] and assessing hospital support for nursing research [30]. Others focused on the global outreach of Italian research and described Italian nurses’ publications in international journals [2] and compared publications in international journals to those in Italian journals [31].

Despite the number of nursing publications having increased considerably in recent years [22], a comprehensive study on the whole of Italian nursing scientific production after 2010 has not been conducted. Furthermore, approaches that focused on academia [22,23,24,25,26,27,28] excluded, by design, both non-academic nurse researchers and fixed-term researchers. Other approaches were not able to draw a complete picture due to their focus on international journals, their limited use of scientific databases [2], and their concern with clinical practice [29] and specific hospitals [30]. A comprehensive study could help to understand Italian nursing scientific production and its strong and weak points, both in academia and in clinical practice, identifying possible strategies to support and strengthen its development. Therefore, the aim of this study was to explore Italian nurses’ publications from 1980 to 2020.

## 2. Materials and Methods

As the global guidelines specifically designed for the reporting of bibliometric analyses are not yet definitively available, the authors of this review followed the recommendations contained in the Preferred Reporting Items for Systematic Review Protocols and Meta-Analyses (PRISMA) [32]. A bibliometric analysis was conducted through a retrospective descriptive study. Several approaches to classifying bibliographic material exist in the literature [33]. Bibliometrics is one of the most common quantitative approaches for analyzing and evaluating the characteristics of a body of literature [33,34,35]. Such analysis makes use of a wide range of indicators measuring the quality of publications, such as type and number of publications, funding, collaborations, etc. For the purposes of the present study, all articles published by Italian nurses from 1980 to 2020 and indexed in Scopus were selected. All scientific articles published in English or Italian whose authors included an Italian nurse with an Italian affiliation were included. All non-scientific articles, as well as conference proceedings, editorials, letters to editors, and books; articles unrelated to nursing; and those for which the full text was not available were excluded.

### 2.1. Data Extraction

**Figure 1:**
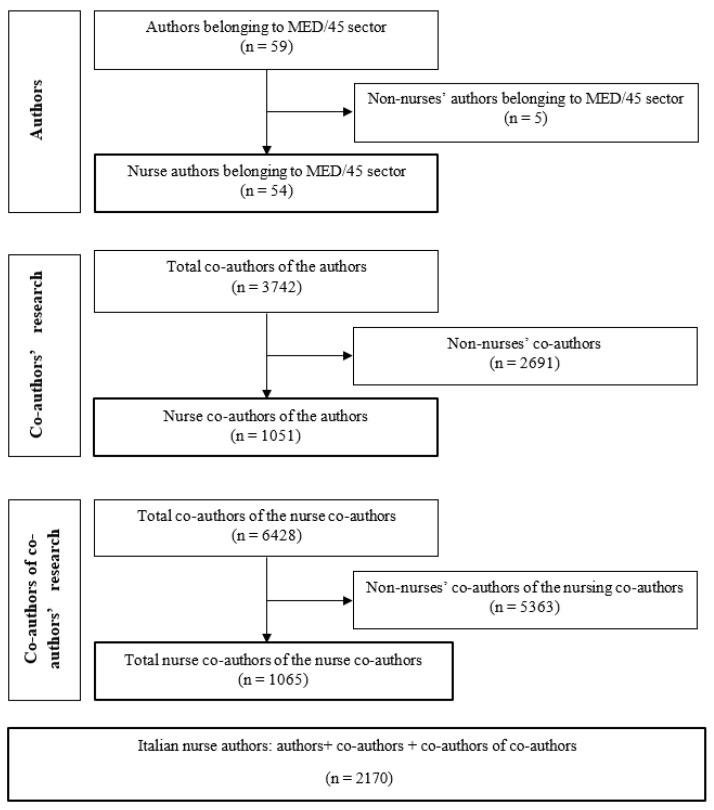
Flow diagram of author selection.

Data were collected through a rigorous process involving several steps (Figure 1). The first step involved consulting, on 18 November 2020, the list of academics (full professors, associate professors, researchers, and research fellows) for the Academic Discipline of General, Clinical and Pediatrics Nursing Sciences published by the Ministry of University and Research [19], which includes 59 people, nurses and non-nurses, in total. The second step involved searching the names of the 59 individuals as authors in Scopus to identify all their co-authors. This was performed via the Scopus “Co-authors” tab in the authors’ profile pages, yielding a total of n = 3742 co-authors, nurses and non-nurses. Then, we repeated this last step by searching each of the co-author’s names in Scopus (n = 3742) and identified the co-authors of the co-authors (n = 6428), nurses and non-nurses. At each step, to identify if the authors or co-authors were nurses, each name was checked for the individual’s professional registration status against the dedicated website for professional registration verification of the National Federation of Orders of Nursing Professions [36]. Non-registered subjects were excluded from the study. In case of homonymy or uncertainty, we searched the internet to find a curriculum vitae or any other official document that could prove whether the author was a nurse. The final number of Italian nurses identified as authors was 2170 (Figure 1).

Each Italian nursing author (n = 2170) was searched in Scopus, and all their published and indexed products were extracted. The records were inputted into Zotero [37], where duplicates and all non-article records, such as books, letters to editors, and conference proceedings were removed. Data extraction ended in December 2020.

### 2.2. Data Analysis

To analyze the identified publications, a classification matrix of the articles was constructed containing qualitative and quantitative information relating to journal, impact factor, language, nursing topic, disciplinary area, type of study, and endogenous vs. exogenous research. ‘Endogenous research’ refers to research that deals with problems and issues concerning nursing care as a profession; ‘exogenous research’ refers to research that investigates and deals with problems and issues centered on patient care [35].

## 3. Results

**Figure 2:**
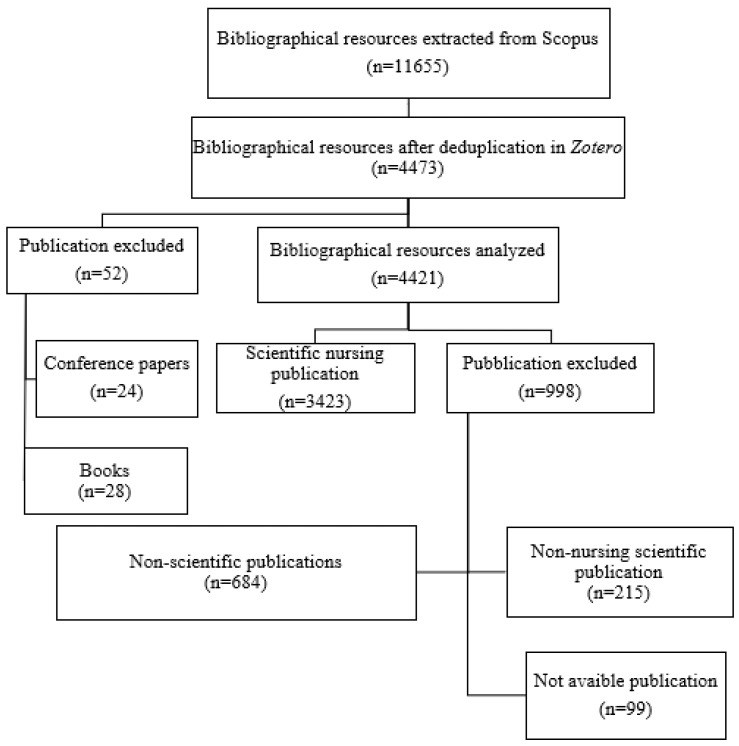
Flow diagram of bibliographical resource extraction.

The search was conducted on a total of n = 2170 Italian nurses, from which n = 11,665 resources were extracted. Following the application of the inclusion and exclusion criteria described in the Materials and Methods section, n = 3423 scientific publications were identified to be analyzed (Figure 2) (Supplementary Materials S1, Analysis of Reports up to 2020).

The trend for scientific publications of Italian nurses showed almost constant growth in the years investigated. Most of the publications were about nursing research (n = 2714, 79.3%). There was an appreciable number of articles on clinical research conducted according to a multidisciplinary approach with other healthcare professionals (n = 709, 20.7%), although the growth of this area was minor.

The scientific publications in nursing research were classified into three main areas. The clinical area (n = 1779) was the most strongly represented, accounting for 51.97% of the scientific publications analyzed, and it also showed the most growth over the years, followed by the education area (n = 485) with 14.17% and the management area (n = 437) with 12.77%, whose trends were more homogeneous. The area designated “other” represented 0.36% (n = 13) and included articles which took a methodological or philosophical approach to the investigation of nursing knowledge.

Regarding national and international collaborations, the research showed a steady increase in international collaborations over the years, from 2 (15.38%) publications produced in collaboration with international authors recorded in the years 1984–1990 to 416 (23.64%) such publications in the last 5 years (2016–2020). Likewise, publications constituting national collaborations have grown significantly and steadily: 11 publications were recorded in the first five-year period (1984–1990), representing 84.62% of publications, and in the last five-year period (2016–2020) 1344 publications (76.36%) were recorded. With respect to national and international collaborations, most scientific publications were concerned with the clinical area, followed by the management and education areas (Table 1).

Italian nurses published for the most part in scientific journals with impact factors (IFs) (94.48%) rather than those without IFs (5.52%). The bibliometric analysis of the IF indicators was carried out by dividing the impact factor values of the scientific journals of the articles extracted into four intervals. The distribution of articles published in journals with an IF in the first interval between 0 and 0,0784 has steadily decreased over time (from 58.52% in the years 2001–2005 to 20.70% in the years 2016–2020), and the distribution in the second interval between 0.0785- and 1.886 increased from 8.15% for the years 2001–2005 to 31.48% for the years 2016–2020. Similarly, the third interval between 1.887 and 2.641 showed a constant growth, from 15.56% in the first five-year period (2001–2005) to 29.26% in the years 2016–2020. The last interval, between 2.642 and 74.669, saw a more dynamic trend, starting from 17.78% in the years 2001–2005 to 18.56% in the last five-year period (2016–2020).

The sources of funding and the studies funded were also analyzed. Most of the research conducted was unfunded (n = 2977, 86.97%), with n = 446 (13.03%) studies being funded. The trend over time shows that funded nursing research steadily increased from 17 publications (7.56%) in the years 2001–2005 to 268 publications (15.23%) in the last five-year period (2016–2020).

Regarding the type of research studies, primary research is the most prevalent type of study (n = 2852), while secondary research (n = 559) is underrepresented. The studies most frequently conducted are quantitative (n = 2473 articles (86.71%)), followed by qualitative studies, with n = 318 articles (11.15%), and mixed-method studies, with n = 61 articles (2.14%) (Table 2 and Table 3).

Finally, the production of endogenous and exogenous scientific research was investigated. Table 3 shows that exogenous, clinically focused research, including patient care, disease management, and educational methods for patients and caregivers, is the most represented, with 1822 articles (67.13%), while endogenous research, referring to aspects of research aimed at nurses and others in the profession with a more management-oriented focus, is represented by 892 articles (32.87%).

## 4. Discussion

The aim of this study was to explore Italian nurses’ publications from 1980 to 2020. We collected research outputs from 2170 Italian nurses, both academic and not, and retrieved 11665 resources. After screening the resources according to inclusion and exclusion criteria and deduplication, we analyzed a total of 3423 articles published by Italian nursing authors. Our findings were not affected by the limitations of previous research, such that we were able to explore all publications by Italian nurses, within academia and without, over a 40-year span. This is relevant because it meant that we were able to provide an overview of the whole Italian nursing research output, its strengths and weaknesses, which can help define the next steps to further improve the quality, quantity, and impact of Italian nursing research.

Overall, we saw an increase in the research output and number of publications in journals with higher IFs, with a leap forward after 2006–2010. This is consistent with the timeline of nursing doctoral programs: it is clear that research production increased after students started achieving PhDs in 2010 [28]. This was important because having a PhD prepared nurses who had a commitment to research and sufficient time and resources to carry it out, and it is imperative to improve nursing research and develop a research culture [38]. Furthermore, having a PhD prepared nurses to improve the research capacity of the country by providing training and supervision to other nurses and healthcare professionals [14]. The increase in IFs could also mean that Italian nursing research, as with nursing research in general, is being increasingly read, used, and cited by national and international colleagues and academics [5], probably due in part to the increased quality of the research itself.

Most of the articles had a clinical focus, followed by educational and administrative foci, with most of them focusing on exogenous research. This is coherent with the domain of nursing research and previous national [22,29] and international studies [6,14,34,39]. In our sample, there were very few studies that focused on methodological or philosophical topics. This might mean that in Italy the philosophical debate is drawn from the international literature rather than published on domestically. However, it could be interesting to further stimulate ontological, epistemological, and axiological discourse in research [40].

Regarding types of study, the majority were primary quantitative studies, with less focus on secondary analysis and qualitative and mixed methodologies. However, when looking at specifics, we noticed that 73% of quantitative studies were observational and that 48% of qualitative studies did not specify which methodology they used. It is recognized that more high-quality experimental and intervention studies and big data analysis are needed in nursing research [39]. However, these types of studies are expensive and time-consuming and need resources and funding to be conducted, which are currently lacking in Italy. With regard to the latter, inexperienced researchers often use qualitative methodologies, being attracted by smaller-scale studies [14]. However, conducting qualitative studies without a methodology, such as “interview studies”, or poorly designed phenomenological studies [41] produces low-quality evidence and continues to harm the respectability of qualitative research [42].

We found an increase in international collaborations over time. Partnerships, collaborations, and networks are considered some of the key interventions to improve research capacity [14]. We also think that, in Italy, collaborations are vital for research due to the chronic nursing faculty shortage. At the time of writing, we have the highest number of academics ever employed in Italy in the Academic Discipline of General, Clinical and Pediatrics Nursing Sciences. In the whole country, there are currently 38 professors (8 full and 30 associate), 18 tenured-track assistant professors, 19 non-tenured-track assistant professors, and 19 post-doctoral fellows [19]. However, they are still too few for a country that has almost 59 million inhabitants [43] and 367.684 nurses [44].

Lastly, we found that, while there has been a slight increase in funding, especially in multidisciplinary research, Italian nursing research is largely unfunded. Unfortunately, this is not only an Italian problem but a European one, as there is not enough government funding available, nor are there sufficient grants specifically intended for nursing research [38,45]. By comparison, in the United States, the National Center for Nursing Research—later called the National Institute for Nursing Research—was established in the 1970s and in the 1980s it already received USD 16.2 million of federal funding [23]. Today, in Europe and Italy, there are only either generic health and medicine grants, or private foundation or society grants. The first are often not available for specific scientific areas of nursing [46] and their reviewers might not be used to evaluating nursing research, while the latter might depend on private-sector availability and research interests. Research funding is believed to be one of the main propellers of nursing research capacity [7,14,29]. For the further development of Italian nursing and nursing research, it is essential to invest in it with specific grants and positions in academia and clinical nursing centers.

### 4.1. Implications for Nursing and Health Policy

Future studies should assess the impact of Italian nursing research and research awareness in clinical practice through quality indicators to evaluate non-bibliometric measures as well. More funding opportunities and nursing research positions are needed.

### 4.2. Limitations and Strengths

Despite all the effort to identify all Italian nurse authors and their publications, we might have missed some. However, we used a rigorous method to retrieve names and verify professional affiliations to overcome the limitations of previous studies. Second, we chose to use only Scopus as our source database, which—by design—meant that we excluded non-indexed publications. However, Scopus is one of the main databases for journal coverage, and it is the chosen database for the Italian National Scientific Qualification, making it the official academic database.

## 5. Conclusions

In this study, we explored Italian nurses’ publications from 1980 to 2020. We analyzed a total of 3423 articles published by 2170 Italian nurses. The overall improvement in the quality and quantity of Italian nursing research is due to the increased presence of PhD-prepared nurses and the number of academics in the country. The clinical area being the focus of publications is coherent with the scope of nursing as a discipline. Non-academic research seems to be in a minority; thus, more nurses in clinical or research organizations should be recruited or dedicated to research. To further improve Italian nursing research, funding opportunities and nursing research positions, both in academia and in clinical centers, are required. Future studies should assess the impact of Italian nursing research on research awareness and the practice of nurses in the country and use quality and content indicators to evaluate non-bibliometric measurements.

## Figures and Tables

**Table 1:** Italian nursing research: collaborations and impact factors.

	Collaborations			Impact Factor				
	International	National	Total	0–0.784	0.785–1.886	1.887–2.641	2.642–74.699	Total
Years	N (%)	N (%)	N (%)	N (%)	N (%)	N (%)	N (%)	N (%)
1984–1990	2 (15.38%)	11 (84.62%)	13 (100%)	5 (50.00%)	4 (40.00%)	0 (0%)	1 (10.00%)	10 (100%)
1991–1995	4 (16.67%)	20 (83.33%)	24 (100%)	12 (57.14%)	4 (19.05%)	3 (14.29%)	2 (9.52%)	21 (100%)
1996–2000	9 (10.47%)	77 (89.53%)	86 (100%)	54 (71.05%)	6 (7.89%)	8 (10.53%)	8 (10.53%)	76 (100%)
2001–2005	18 (8.00%)	207 (92.00%)	225 (100%)	79 (58.52%)	11 (8.15%)	21 (15.56%)	24 (17.78%)	135 (100%)
2006–2010	38 (8.50%)	409 (91.50%)	447 (100%)	139 (50.55%)	43 (15.64%)	52 (18.91%)	41 (14.91%)	275 (100%)
2011–2015	133 (15.32%)	735 (84.68%)	868 (100%)	221 (34.16%)	161 (24.88%)	173 (26.74%)	92 (14.22%)	647 (100%)
2016–2020	416 (23.64%)	1344 (76.36%)	1760 (100%)	290 (20.70%)	441 (31.48%)	(410) 29.26%	260 (18.56%)	1401 (100%)
Total	620 (18.11%)	2803 (81.89%)	3423 (100%)	800 (31.19%)	670 (26.12%)	667 (26.00%)	428 (16.69%)	2565 (100%)

**Table 2:** Distribution of research typology.

Distribution of Research Typology	N	(%)
SECONDARY RESEARCH	559	16.33%
PRIMARY RESEARCH	2852	83.32%
MIXED METHOD	61	1.78%
QUALITATIVE STUDY	318	9.29%
Ethnographic	6	1.78%
Phenomenological	67	1.96%
Grounded Theory	14	0.41%
Not Specified	152	4.44%
Other Qualitative	79	2.31%
QUANTITATIVE	2473	72.2%
Experimental Study	427	12.5%
Instrumental Study	230	6.72%
Other Methodology	5	0.15%
Observational Study	1811	52.91%
NOT CLASSIFIABLE	12	0.35%
METHODOLOGICAL ARTICLE	11	0.32%
PHILOSOPHICAL ANALYSIS—THEORETHICAL PAPER	1	0.03%
Total	3423	100%

**Table 3:** Types of research and years of publication for endogenous and exogenous research.

Years of Publication	Mixed Method		Qualitative Study		Quantitative Study		Tot. n°	Tot. %	Endogenous Research		Exogenous Research		Total	
	n	%	n	%	n	%			n	%	n	%	n	%
1984–1990		0.00%		0.00%	12	100.00%	12	100.00%	4	36.36%	7	63.64%	11	100.00%
1991–1995		0.00%	4	22.22%	14	77.78%	18	100.00%	12	52.17%	11	47.83%	23	100.00%
1996–2000		0.00%	16	22.54%	55	77.46%	71	100.00%	25	32.05%	53	67.95%	78	100.00%
2001–2005	1	0.53%	16	8.51%	171	90.96%	188	100.00%	46	31.29%	101	68.71%	147	100.00%
2006–2010	5	1.27%	45	11.45%	343	87.28%	393	100.00%	129	38.28%	208	61.72%	337	100.00%
2011–2015	14	1.93%	77	10.59%	636	87.48%	727	100.00%	229	33.33%	458	66.67%	687	100.00%
2016–2020	41	2.84%	160	11.09%	1242	86.07%	1443	100.00%	447	31.24%	984	68.76%	1431	100.00%
Total	61	2.14%	318	11.15%	2473	86.71%	2852	100.00%	892	32.87%	1822	67.13%	2714	100.00%

## Data Availability

All data produced are included in the article and the Supplementary Materials.

## Supplementary Materials

The following supporting information can be downloaded at: https://www.mdpi.com/article/10.3390/nursrep14040287/s1, File S1: Reports up to 2020.

## Public Involvement Statement

There was no public involvement in any aspect of this research.

## Guidelines and Standards Statement

This manuscript was drafted according to the PRISMA guidelines (Preferred Reporting Items for Systematic Review Protocols and Meta-Analyses [32]) for systematic review research.
